# The Effects of Pre-Existing Hyponatremia and Subsequent-Developing Acute Kidney Injury on In-Hospital Mortality: A Retrospective Cohort Study

**DOI:** 10.1371/journal.pone.0162990

**Published:** 2016-09-13

**Authors:** Sung Woo Lee, Seon Ha Baek, Shin Young Ahn, Ki Young Na, Dong-Wan Chae, Ho Jun Chin, Sejoong Kim

**Affiliations:** 1 Department of Internal Medicine, Seoul National University Postgraduate School, Seoul, Korea; 2 Department of Internal Medicine, Eulji General Hospital, Seoul, Korea; 3 Department of Internal Medicine, Seoul National University Bundang Hospital, Seongnam, Korea; 4 Department of Internal Medicine, Korea University Guro Hospital, Seoul, Korea; University of Sao Paulo Medical School, BRAZIL

## Abstract

**Background and Objectives:**

Both hyponatremia and acute kidney injury (AKI) are common and harmful in hospitalized patients. However, their combined effects on patient mortality have been little studied.

**Methods:**

We retrospectively enrolled 19191 adult patients who were admitted for 1 year. Pre-existing hyponatremia was defined as a serum sodium level < 135 mmol/L on the first measurement of their admission. AKI was defined as a rise in serum creatinine by ≥ 26.5 μmol/L or ≥ 1.5 times of the baseline value of creatinine during the hospital stay.

**Results:**

The prevalence of pre-existing hyponatremia was 8.2%. During a median 6.0 days of hospital stay, the incidence rates of AKI and in-hospital patient mortality were 5.1% and 0.9%, respectively. Pre-existing hyponatremia independently predicted AKI development and in-hospital mortality (adjusted hazard ratio [HR] 1.300, *P* = 0.004; HR 2.481, *P* = 0.002, respectively). Pre-existing hyponatremia and subsequent development of AKI increased in-hospital mortality by 85 times, compared to the patients with normonatremia and no AKI. In subgroup analysis, the AKI group showed higher rates of de novo hypernatremia than the non-AKI group during the admission. De novo hypernatremia, which might be associated with over-correction of hyponatremia, increased in-hospital mortality (HR 3.297, *P* <0.001), and patients with AKI showed significantly higher rates of de novo hypernatremia than patients without AKI (16.2% vs. 1.4%, *P* < 0.001, respectively).

**Conclusion:**

Pre-existing hyponatremia may be associated with the development of AKI in hospitalized patients, and both hyponatremia and hospital-acquired AKI could have a detrimental effect on short term patient mortality, which might be related to the inappropriate correction of hyponatremia in AKI patients.

## Introduction

Hyponatremia is the most common disorder of body fluid and electrolyte balance in hospitalized patients [1]. The presence of hyponatremia is hazardous since it is associated with increased risk of mortality in hospitalized patients [2], including those with heart [3] and liver [4] diseases, stroke [5], and chronic kidney disease (CKD) [6, 7]. According to recent meta-analysis, hyponatremic subjects showed a 2.6 times higher risk of death than non-hyponatremic subjects, even when the decrease in the serum sodium level was moderate [8].

Acute kidney injury (AKI) is also a common, but harmful disease [9]. As with hyponatremia, the presence of AKI is dangerous since it predicts mortality in critically or non-critically ill hospitalized patients [10, 11] and in community based patients [12]. There are several causes of AKI such as volume depletion [13] or overload [14, 15], sepsis [16], and heart [17] and liver [18] disease. Hyponatremia is also frequently developed in conditions with volume imbalances which can provoke the development of AKI [1, 19, 20]. Once AKI is developed, the chances of fluid overload and overcorrection of hyponatremia are increasing [21, 22], and thus extra-cautions for the treatment of electrolyte imbalance are warranted.

Under specific conditions such as cancer, cardiorenal syndrome, and liver transplantation, hyponatremia has been correlated with the development of AKI [23–25]. In contrast, cross-talk between pre-existing hyponatremia and hospital acquired AKI in a large cohort study has been sparsely investigated [26, 27]. Therefore, we investigated whether pre-existing hyponatremia could predict the development of AKI, and whether AKI and hyponatremia have a combined effect on patient mortality using data from an 1-year administrative cohort.

## Material and Methods

### Patients

**Fig 1:**
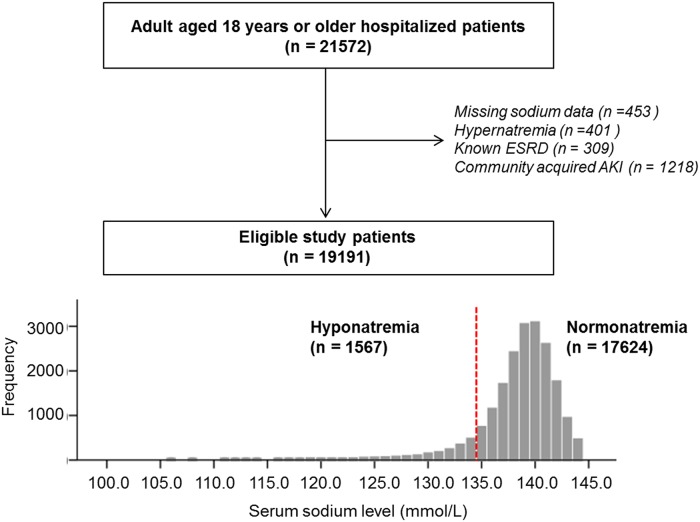
Algorithm for eligible patient selection. Hypernatremia is defined as sodium 145 mmol/L or higher. ESRD, end stage renal disease; AKI, acute kidney injury.

We collected data from 21572 adult patients aged 18 years or older, whose serum creatinine was identified once or more during their first admission for 1 year in 2013 at a tertiary care hospital, Seongnam, Korea (Fig 1). Among these, 19592 patients were eligible for the study after excluding patients with missing sodium data (n = 453), known end stage renal disease (ESRD, n = 309) and community acquired AKI (n = 1218). Of the remaining 19592 patients, we only included 19191 patients after further excluding 401 patients with hypernatremia at admission since hypernatremia is another independent risk factor of mortality [28] and is beyond the scope of this study. The study protocol complies with the Declaration of Helsinki and received full approval from the Seoul National University Hospital`s institutional review board (IRB number: B-1511/322-112), which waived the need for informed consent since the study did not infringe on patient privacy or health status.

### Definitions and measurements

Demographic, physiological, and laboratory data at the index admission were gathered from the electronic medical records database. After different patient datasets were merged, data verification was performed manually. Hypertension was defined as the presence of a physician diagnosis or receiving anti-hypertensive drugs. Anti-hypertensive drugs included alpha and beta blockers, calcium channel blockers, renin angiotensin system (RAS) inhibitors, and diuretics. Diabetes was defined as physician diagnosis or being prescribed insulin or oral anti-diabetic drugs. Cardiovascular disease was defined as a physician diagnosis of angina, ischemic heart disease, myocardial infarction or cerebrovascular disease. Body mass index was calculated as weight (kg) per square of height (m^2^). Biochemistry analysis including serum glucose was performed using random blood or urine samples. For the inflammatory markers, we used white blood cell counts, red cell distribution width (RDW) [11], neutrophil to lymphocyte ratio (NLR) [29], and C-reactive protein (CRP).

Using the first serum sodium level of the index admission, we defined hypo- and hypernatremia as serum sodium < 135 mmol/L [1] and ≥ 145 mmol/L [30], respectively. Among 19191 patients, 18353 had serial data of serum sodium levels during their index admission. De novo hypernatremia was defined as at least one event of hypernatremia during the hospital stay. Corrected hyponatremia was defined as mean serum sodium level during hospital stay ≥ 135 mmol/L in hyponatremic patients. De novo hyponatremia was defined as cases with mean serum sodium level during hospital stay < 135 mmol/L in normonatremic patients. When mean serum sodium levels were ≥ 135 mmol/L in normonatremic patients and < 135 mmol/L in hyponatremic patients, we defined these as persistent normonatremia and hyponatremia, respectively.

Serum creatinine was measured using the rate-blanked compensated kinetic alkaline picrate Jaffe method with an automatic analyzer (Toshiba-200FR, Tokyo, Japan). The between-day coefficients of variation for serum creatinine were 1.5–2.6% and 1.1–2.4% at low (168.0–176.8 μmol/L), and high concentrations (583.4–627.6 μmol/L), respectively, throughout the study periods. The estimated glomerular filtration rate (eGFR) was calculated by using the equation of the Chronic Kidney Disease Epidemiology Collaboration [31]. Baseline creatinine was defined as the lowest value within 6 months before the index admission or the calculated value from the Modification of Diet in Renal Disease study equation, assuming GFR 75 ml/min/1.73 m^2^ if creatinine was not available [32]. AKI was defined as a rise in serum creatinine by ≥ 26.5 mg/dL, or ≥ 1.5 times from the baseline level determined during the hospital stay [33, 34]. We classified a rise in serum creatinine by ≥ 2 times from the baseline as severe AKI. If the first creatinine of the index admission met the criteria for AKI, we defined it as a community acquired AKI. In-hospital patient mortality was determined by whether a death certificate had been issued at 90 days after admission. ESRD development was determined from the registry database of the Korean Society of Nephrology, and 1 year-mortality was determined from the database of Ministry of the interior.

### Statistical analysis

Values were expressed as mean ± SD or median (interquartile range, IQR) for continuous variables and percentage for categorical variables. The difference was analyzed by Student`s *t* test for continuous variables and the chi-square test for categorical variables. *P*-trend was assessed by a linear-by-linear association. *P* < 0.05 was considered to have statistical significance. For the estimated survival, the Kaplan-Meier method was employed, and the statistical significance was calculated using the log-rank test. The risk of a certain factor for AKI or in-hospital mortality was assessed using Cox proportional hazard regression analysis reporting hazard ratio (HR) and its confidence interval (CI). The assumption of proportional hazards was tested by log minus log plot for categorical variables and interaction analysis with time covariate using time-dependent Cox regression for continuous variables. All covariates entered into the model fulfilled the assumption. Since known risk factors for AKI or mortality were generally statistically significant in this study, we entered variables with *P* < 0.05 in univariate analysis and missing rate < 15% into the multivariate modeling to avoid over-adjustment. The multiplicative interaction was evaluated by entering the interaction term as a covariate in logistic regression analysis. The additive interaction was assessed by Relative Excess Risk due to Interaction (RERI), Attributable Proportion due to interaction (AP) and Synergistic Index (SI) [35] using R statistics (Version 3.0.3, R foundation for Statistical Computing Platform). Unless specified, all analysis was performed using SPSS Statistics (version 22; IBM, USA).

## Results

Of 19191 patients, the mean age was 58.1 ± 17.0 years and 52.4% were men. In total, 17.8% had hypertension, 20.3% had diabetes, 13.5% had cardiovascular disease, and 24.8% had cancer. In this population, 8.2% were hyponatremic initially. During a median (IQR) 6.0 (3.0–10.0) days of hospital stay, 5.1% experienced AKI of all stages, and 0.9% died from all causes within 90 days after admission. No patient died before the development of AKI. Among patients with the development of AKI or in-hospital mortality, the median (IQR) duration from the admission date to the development of AKI and in-hospital mortality were 5.0 (2.0–10.0) days and 13.6 (6.7–27.3) days, respectively.

### Characteristics of hyponatremia and AKI group

We compared baseline characteristics according to the status of hyponatremia (Table 1). Older age and male were associated with hyponatremia. Patients with hyponatremia showed higher proportions of hypertension, diabetes, and cancer, and a lower proportion of cardiovascular disease than those with normonatremia. RAS inhibitors and diuretics use were associated with hyponatremia. Hyponatremic patients had lower body mass index, systolic and diastolic blood pressure (BP) and higher serum glucose than normonatremic patients. Patients with hyponatremia revealed significantly higher levels of inflammation, as indicated by white blood cells, NLR, RDW, and CRP, than those with normonatremia. Hemoglobin levels were significantly lower in the hyponatremic group than in the normonatremic group. Hyponatremic patients had lower eGFRs than normonatremic patients.

**Table 1:** Baseline characteristics of sodium group.

	Normonatremia (n = 17624)	Hyponatremia (n = 1567)	*P*
Age (years)	57.4 ± 16.9	65.4 ± 16.3	< 0.001
Male sex	9156 (52.0)	908 (57.9)	< 0.001
Hypertension	3022 (17.1)	395 (25.2)	< 0.001
Diabetes	3317 (18.8)	570 (36.4)	< 0.001
Cardiovascular disease	2431 (13.8)	165 (10.5)	< 0.001
Congestive heart failure	147 (0.8)	27 (1.7)	< 0.001
Liver disease	1061 (6.0)	143 (9.1)	< 0.001
Cancer	4229 (24.0)	538 (34.3)	< 0.001
RAS inhibitor	1210 (6.9)	166 (10.6)	< 0.001
Diuretics	705 (4.0)	162 (10.3)	< 0.001
Body mass index (kg/m^2^)*	23.9 ± 3.6	22.3 ± 4	< 0.001
Systolic BP (mmHg)*	130.7 ± 20.5	127.3 ± 22.3	< 0.001
Diastolic BP (mmHg)*	75.6 ± 13.5	71.8 ± 14	< 0.001
Sodium (mmol/L)	139.4 ± 2.2	131.2 ± 3.6	< 0.001
Glucose (mmol/L)*	6.9 ± 2.6	8.6 ± 5.2	< 0.001
White blood cells (10^3^/μL)*	8.2 ± 5.5	9.8 ± 7.2	< 0.001
Hemoglobin (g/dL)*	12.8 ± 2	11.7 ± 2.2	< 0.001
Platelet (10^3^/μL)*	217.5 ± 77.6	223.8 ± 105.5	0.021
NLR*	4.8 ± 5.2	7.4 ± 7.9	< 0.001
RDW (%)*	13.6 ± 1.7	14.2 ± 2.2	< 0.001
C-reactive protein (nmol/L)*	467.6 ± 567.5	717.6 ± 697.8	< 0.001
Protein (g/L)*	65.7 ± 7.9	63.4 ± 8.8	< 0.001
Albumin (g/L)*	39.7 ± 5.7	35.2 ± 6.2	< 0.001
Cholesterol (mmol/L)*	4.5 ± 1.2	4.1 ± 1.5	< 0.001
Bilirubin (μmol/L)*	13.8 ± 19.6	22.2 ± 43.1	< 0.001
Blood urea nitrogen (mmol/L)*	5.3 ± 2.5	6.0 ± 3.6	< 0.001
Serum creatinine (μmol/L)	78.4 ± 32.8	80.1 ± 55.6	0.248
eGFR (ml/min/1.73m^2^)	86.3 ± 18.5	83.8 ± 22.5	< 0.001

RAS, renin angiotensin system; BP, blood pressure; NLR, neutrophil to lymphocyte ratio; RDW, red cell distribution width; eGFR, estimated glomerular filtration rate.

Values are expressed as mean ± standard deviation for continuous variables and n (%) for categorical variables. Comparisons were made by chi square for categorical variables or *Student t*-test for continuous variables.

* Incomplete data. The missing data rate was 9.4% in body mass index, 0.3% in systolic and diastolic BP, 12.2% in glucose, 1.1% in white blood cells, hemoglobin and platelet, 30.2% in NLR and RDW, 44.4% in C-reactive protein, 2.2% in protein, 1.5% in albumin, 2.1% in cholesterol, 1.5% in bilirubin and 0.2% in blood urea nitrogen.

We also explored factors associated with AKI development (Table 2). Older age, male, and the presence of comorbidities were related to AKI development. RAS inhibitors and diuretics were associated with AKI development. Patients with AKI showed higher levels of serum glucose and inflammation than those without AKI. The AKI group had significantly lower levels of hemoglobin than the non-AKI group. Higher levels of blood urea nitrogen (BUN) were associated with AKI development. In multivariate Cox proportional hazard regression analysis, male, diabetes, congestive heart failure, diuretics use, diastolic BP, and levels of hemoglobin, protein, albumin, bilirubin, and BUN were associated with AKI development.

**Table 2:** Factors associated with acute kidney injury development.

	Univariate			Multivariate†	
	Non-AKI (n = 18215)	AKI (n = 976)	*P*	HR (95% CI)	*P*
Age (years)‡	57.7 ± 17.0	65.3 ± 15.6	< 0.001	1.118 (0.954–1.310)	0.168
Male sex	9501 (52.2)	563 (57.7)	0.001	1.262 (1.088–1.465)	0.002
Hypertension	3148 (17.3)	269 (27.6)	< 0.001	1.015 (0.822–1.253)	0.891
Diabetes	3449 (18.9)	438 (44.9)	< 0.001	1.885 (1.622–2.192)	< 0.001
Cardiovascular disease	2445 (13.4)	151 (15.5)	0.068	-	-
Congestive heart failure	129 (0.7)	45 (4.6)	< 0.001	2.986 (2.072–4.303)	< 0.001
Liver disease	1136 (6.2)	68 (7.0)	0.359	-	-
Cancer	4443 (24.4)	324 (33.2)	< 0.001	1.088 (0.935–1.265)	0.275
RAS inhibitor	1269 (7.0)	107 (11.0)	< 0.001	1.026 (0.786–1.340)	0.850
Diuretics	754 (4.1)	113 (11.6)	< 0.001	1.514 (1.151–1.993)	0.003
Body mass index (kg/m^2^)*	23.8 ± 3.7	23.2 ± 4.0	< 0.001	1.000 (0.982–1.018)	0.991
Systolic BP (mmHg)*	130.4 ± 20.4	130.9 ± 25.0	0.562	-	-
Diastolic BP (mmHg)*	75.4 ± 13.5	74 ± 15.2	0.006	1.006 (1.001–1.011)	0.016
Hyponatremia	1366 (7.5)	201 (20.6)	< 0.001	1.300 (1.086–1.555)	0.004
Sodium (mmol/L)	138.8 ± 3.1	137.1 ± 4.7	< 0.001	-	-
Glucose (mmol/L)*‡	7.0 ± 2.9	8.0 ± 4.1	< 0.001	1.030 (0.890–1.192)	0.695
WBC (10^3^/μL)*	8.3 ± 5.5	9.5 ± 7.7	< 0.001	1.006 (0.997–1.014)	0.185
Hemoglobin (g/dL)*	12.8 ± 2.0	11.8 ± 2.3	< 0.001	0.959 (0.921–0.998)	0.038
Platelet (10^3^/μL)*‡	218.2 ± 78.6	214.2 ± 105.6	0.247	-	-
NLR*‡	4.9 ± 5.3	7.3 ± 8.7	< 0.001	-	-
RDW (%)*	13.6 ± 1.8	14.3 ± 1.9	< 0.001	-	-
CRP (nmol/L)*	482.9 ± 578.5	628.0 ± 674.2	< 0.001	-	-
Protein (g/L)*	65.6 ± 7.9	63.1 ± 9.0	< 0.001	1.013 (1.003–1.023)	0.012
Albumin (g/L)*	39.5 ± 5.8	35.8 ± 6.6	< 0.001	0.948 (0.933–0.964)	< 0.001
Cholesterol (mmol/L)*‡	4.5 ± 1.2	4.1 ± 1.4	< 0.001	1.072 (0.917–1.253)	0.385
Bilirubin (μmol/L)*	14.0 ± 19.9	23.7 ± 50.5	< 0.001	1.003 (1.001–1.004)	< 0.001
BUN (mmol/L)*‡	5.3 ± 2.5	7.0 ± 4.5	< 0.001	1.559 (1.352–1.798)	< 0.001
Serum creatinine (μmol/L)	78.3 ± 31.3	82.7 ± 78.1	0.082	-	-
eGFR (ml/min/1.73m^2^)‡	86.1 ± 18.2	86.0 ± 28.9	0.201	-	-
eGFR ≥ 86.1 ml/min/17.3m^2^^$^	7725 (42.4)	430 (44.1)	0.310	-	-
eGFR (min.–max.)	2.4–210.4	3.4–193.2	-	-	-

HR, hazard ratio; CI, confidence interval; RAS, renin angiotensin system; BP, blood pressure; WBC, white blood cells; NLR, neutrophil to lymphocyte ratio; RDW, red cell distribution width; CRP, c-reactive protein; BUN, blood urea nitrogen; eGFR, estimated glomerular filtration rate.

Values are expressed as mean ± standard deviation for continuous variables and n (%) for categorical variables. Comparisons were made by chi-square test for categorical variables or *Student t*-test for continuous variables. HRs for categorical and continuous variables were “yes vs. no” and “per 1 unit increase”, respectively.

* Incomplete data. The missing data rate was 9.4% in body mass index, 0.3% in systolic and diastolic BP, 12.2% in glucose, 1.1% in white blood cells, hemoglobin and platelet, 30.2% in NLR and RDW, 44.4% in CRP, 2.2% in protein, 1.5% in albumin, 2.1% in cholesterol, 1.5% in bilirubin and 0.2% in BUN.

^†^Cox proportional hazard regression analysis entering variables with *P* < 0.05 in univariate analysis and missing rate < 15%.

^‡^ Not fulfilling the proportional hazard assumption as continuous variables, but fulfilling the assumption as categorical variables by their mean value in whole patients: 58.7 years for age, 7.1 mmol/L for glucose, 5.3 for NLR, 4.4 mmol/L for cholesterol, and 6.0 mmol/L for BUN. The reported HRs were above vs. below the mean.

^$^ Mean of eGFR in whole population.

### Hyponatremia, AKI and mortality

**Fig 2:**
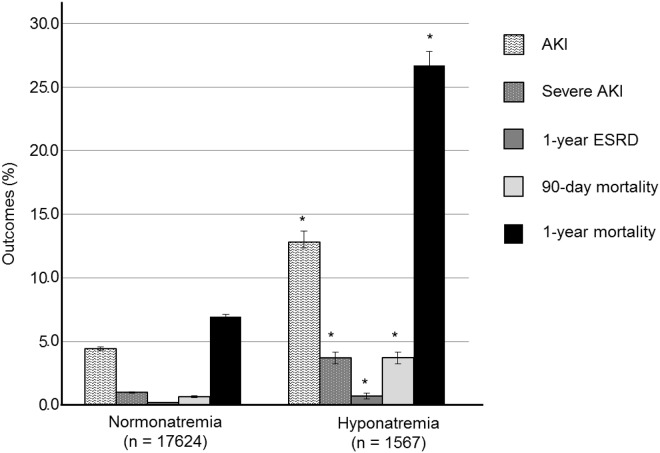
Clinical outcomes according to sodium group. * means *P* < 0.001 compared to normonatremia group. Error bar indicates standard error. AKI, acute kidney injury; ESRD, end stage renal disease.

Hyponatremic patients showed significantly higher incidence rates of AKI than normonatremic patients (Fig 2). Patients with AKI had considerably lower serum sodium levels than patients without AKI (Table 2). After adjusting for covariates including age, male, hypertension, diabetes, congestive heart failure, cancer, RAS inhibitors, diuretics, body mass index, diastolic BP, white blood cells, hemoglobin, and serum levels of glucose, protein, albumin, cholesterol, bilirubin, and BUN, hyponatremia was independently associated with the increased risk of AKI development with HR 1.300 (95% CI, 1.086–1.555; *P* = 0.004) in multivariate Cox proportional hazard regression.

**Fig 3:**
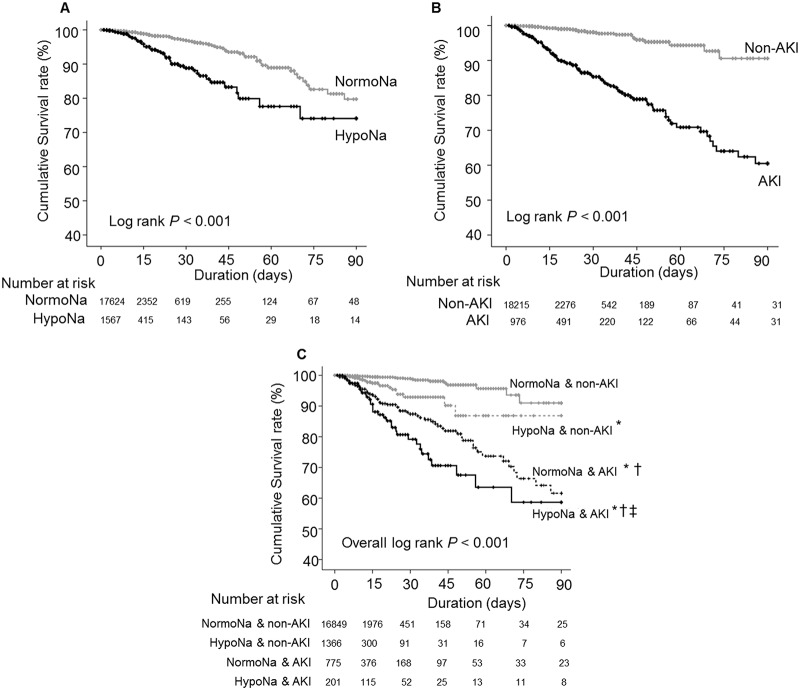
Cumulative survival rate according to sodium (Na) and acute kidney injury (AKI) groups. A, B and C show survival curves of sodium, AKI and combined sodium and AKI groups for the in-hospital mortality, respectively. * and † indicate *P* < 0.001 when compared to normonatremic patients without AKI and hyponatremic patients without AKI groups, respectively, and ‡ indicates *P* < 0.05 when compared to normonatremic with AKI group using log-rank test.

Hyponatremic patients had considerably poorer patient survival rates than normonatremic patients (Fig 3A). Patients with AKI also showed significantly shorter patient survival times than those without AKI (Fig 3B). Both hyponatremia and AKI were significantly related to the increased risk of in-hospital mortality with HRs (95% CI) of 2.481 (1.381–4.459, *P* = 0.002) and 7.338 (4.727–11.391, *P* < 0.001) in multivariate Cox proportional hazard regression analysis (Table 3). Since hyponatremia was related to AKI development, we explored the interaction between hyponatremia and AKI for the development of in-hospital mortality (Table 4). Compared to the patients with normonatremia and no AKI, patients with pre-exisiting hyponatremia and patients with AKI had worse in-hospital mortality (OR 7.515, *P* < 0.001; OR 45.69, *P* < 0.001, respectively) (Table 4). Pre-existing hyponatremia and subsequent development of AKI increased the risk of short-term morality by 85.55 times, compared to the controls, although not statistically significant in RERI. We compared patient survival for different sodium levels and the presence of AKI (Fig 3C).

**Table 3:** Hazard ratio for in-hospital mortality in Cox proportional hazard regression.

	Univariate		Multivariate	
	HR (95% CI)	*P*	HR (95% CI)	*P*
Age (years)	1.044 (1.032–1.056)	< 0.001	1.025 (1.011–1.039)	< 0.001
Male sex	1.339 (0.987–1.818)	0.061	-	-
Hypertension	1.742 (1.262–2.405)	0.001	1.034 (0.627–1.707)	0.895
Diabetes	2.351 (1.731–3.192)	< 0.001	1.140 (0.793–1.639)	0.479
Cardiovascular disease	2.177 (1.540–3.077)	< 0.001	2.209 (1.428–3.416)	< 0.001
Congestive heart failure	3.801 (1.869–7.729)	< 0.001	0.743 (0.285–1.939)	0.544
Liver disease	1.106 (0.565–2.165)	0.768	-	-
Cancer	1.654 (1.225–2.233)	0.001	1.916 (1.331–2.758)	< 0.001
RAS inhibitor	1.751 (1.087–2.820)	0.021	0.915 (0.500–1.674)	0.774
Diuretics	3.293 (2.155–5.031)	< 0.001	1.543 (0.829–2.873)	0.171
BMI ≥23.7 kg/m^2^†	0.393 (0.270–0.571)	<0.001	0.500 (0.341–0.734)	< 0.001
SBP ≥130.2 mmHg†	0.659 (0.485–0.897)	0.008	0.666 (0.455–0.975)	0.037
DBP ≥74.9 mmHg†	0.627 (0.462–0.852)	0.003	1.138 (0.784–1.652)	0.497
Glucose (mmol/L)	1.072 (1.042–1.103)	< 0.001	1.007 (0.965–1.051)	0.746
WBC (10^3^/μL)	1.020 (1.013–1.026)	< 0.001	1.017 (1.007–1.027)	0.001
Hemoglobin (g/dL)	0.849 (0.796–0.906)	< 0.001	1.047 (0.959–1.143)	0.308
Platelet (10^3^/μL)	0.996 (0.994–0.998)	< 0.001	0.998 (0.996–0.999)	0.009
NLR*	1.037 (1.028–1.046)	< 0.001	-	-
RDW (%)*	1.232 (1.174–1.293)	< 0.001	-	-
CRP (nmol/L)*	1.001 (1.001–1.001)	< 0.001	-	-
Protein (g/L)	0.964 (0.949–0.979)	< 0.001	1.005 (0.984–1.026)	0.625
Albumin ≥ 39 g/L†	0.290 (0.200–0.420)	<0.001	0.456 (0.279–0.746)	0.002
Cholesterol (mmol/L)	0.664 (0.580–0.759)	< 0.001	0.869 (0.757–0.997)	0.045
Bilirubin (μmol/L)	1.003 (1.001–1.006)	0.004	1.002 (1.000–1.005)	0.088
BUN ≥ 6.0 mmol/L †	2.730 (2.009–3.708)	<0.001	1.301 (0.915–1.850)	0.143
eGFR (ml/min/1.73m^2^)	0.993 (0.987–1.000)	0.051	-	-
Hyponatremia	2.915 (2.121–4.006)	< 0.001	2.481 (1.381–4.459)	0.002
AKI	8.711 (6.273–12.097)	< 0.001	7.338 (4.727–11.391)	< 0.001

HR, hazard ratio; CI, confidence interval; RAS, renin angiotensin system; BP, blood pressure; WBC, white blood cells; NLR, neutrophil to lymphocyte ratio; RDW, red cell distribution width; CRP, c-reactive protein; BUN, blood urea nitrogen; eGFR, estimated glomerular filtration rate; AKI, acute kidney injury.

HRs for categorical and continuous variables were “yes vs. no” and “per 1 unit increase”, respectively. Interaction term between hyponatremia and AKI was statistically significant in both univariate (*P* = 0.001) and multivariate (*P* = 0.024) analysis. Variables with *P* < 0.05 in univariate analysis and missing rate < 15.0% were entered into multivariate analysis.

* Missing rate ≥15.0%

^†^ Not fulfilling the proportional hazard assumption as continuous variables, but fulfilling the assumption as categorical variables by their mean value in whole patients.

**Table 4:** Interaction analysis between hyponatremia and acute kidney injury for the in-hospital mortality.

	AKI				OR (95% CI) for AKI (yes vs. no) within strata of sodium group
	No		Yes		
Hyponatremia	N*	OR (95% CI)	N*	OR (95% CI)	
No	40/16809	1.0 (reference)	76/699	45.690 (30.926–67.501)	45.690 (30.926–67.501)
Yes	24/1342	7.515 (4.517–12.504)	34/167	85.554 (52.838–138.529)	11.384 (6.590–19.667)
OR (95% CI) for hyponatremia (yes vs. no) within strata of AKI group		7.515 (4.517–12.504)		1.873 (1.208–2.902)	

Measure of interaction on additive scale: RERI (95% CI) 33.350 (-1.611–68.311), AP (95% CI) 0.390 (0.129–0.650) and SI (95% CI) 1.651 (1.071–2.546); Measure of interaction on multiplicative scale: OR (95% CI) 0.249 (0.127–0.488). ORs are unadjusted. OR, odd ratio; CI, confidence interval; AKI, acute kidney injury; RERI, relative excess risk due to interaction; AP, attributable proportion due to interaction; SI, synergistic index.

*with/without in-hospital mortality

The hyponatremia & non-AKI, normonatremia & AKI, and hyponatremia & AKI groups showed significantly poorer patient survival than the normonatremia & non-AKI group. This was still evident in multivariate Cox proportional hazard regression analysis adjusting for the same variables in Table 3. HRs (95% CI) for the hyponatremia & non-AKI, normonatremia & AKI, and hyponatremia & AKI groups vs. the normonatremia & non-AKI group for the in-hospital mortality were 2.481 (1.381–4.458, *P* = 0.002), 7.338 (4.727–11.391, *P* < 0.001) and 7.951 (4.677–13.515, *P* < 0.001), respectively.

**Fig 4:**
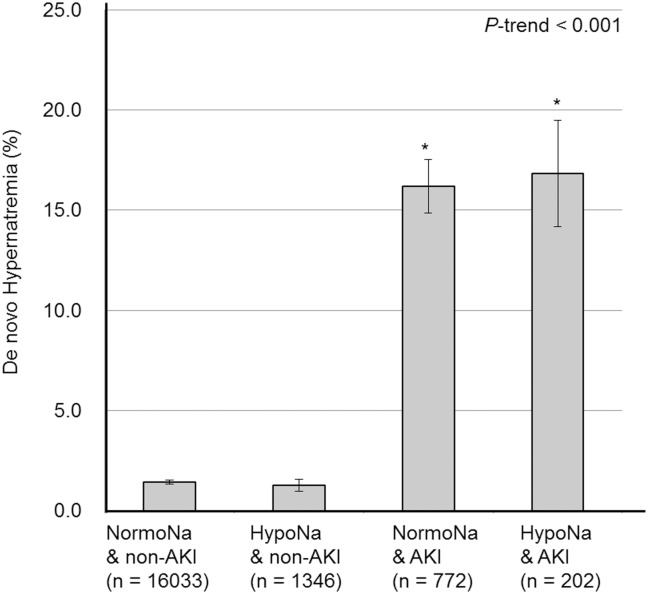
Proportion of de novo hypernatremia depending on the status of combined sodium (Na) and acute kidney injury (AKI) group. Error bar indicates standard error. * indicates *P* < 0.05 by chi-square test when compared to the normoNa & non-AKI group.

As one of the overcorrection variables, we evaluated de novo hypernatremia. Of 18353 patients who had serial data of serum sodium levels, we found 405 patients with de novo hypernatremia (2.1%). De novo hypernatremia was also significantly associated with in-hospital mortality with HR 3.297 (95% CI 2.264–4.800, *P* <0.001) in a multivariate Cox proportional hazard regression model. Meanwhile, patients with AKI (16.2%) showed significantly higher rates of de novo hypernatremia than patients without AKI (1.4%; *P* < 0.001). Through examining different combinations of sodium levels and AKI, we identified a significant tendency of an increased rate of de novo hypernatremia across the normonatremia & non-AKI, hyponatremia & non-AKI, normonatremia & AKI and hyponatremia & AKI groups (Fig 4).

Hyponatremic patients also showed a significantly higher risk of severe AKI, 1-year ESRD, and 1-year mortality than normonatremic patients (Fig 2).

### Effect of hyponatremia correction

Of 18353 patients who had serial data on serum sodium levels, 84.5%, 3.1%, 2.2% and 5.9% were classified as persistent normonatremia, de novo hyponatremia, corrected hyponatremia, and persistent hyponatremia groups, respectively. Patients with persistent normonatremia showed consistently lower risks of AKI development and in-hospital mortality than those with corrected hyponatremia. Patients with de novo hyponatremia still had a lower risk of in-hospital mortality than those with corrected hyponatremia. The risks of AKI development and in-hospital mortality were not different between patients with persistent and corrected hyponatremia (Table 5).

**Table 5:** Hazard ratio of hyponatremia correction group for the development of acute kidney injury and in-hospital mortality in multivariate Cox proportional hazard regression analysis.

	For AKI development		For in-hospital mortality	
	HR (95% CI)	*P*	HR (95% CI)	*P*
vs. Corrected hyponatremia				
Persistent normonatremia	0.606 (0.457–0.803)	< 0.001	0.514 (0.312–0.846)	0.009
De novo hyponatremia	0.894 (0.643–1.242)	0.505	0.504 (0.264–0.964)	0.038
Persistent hyponatremia	0.793 (0.578–1.088)	0.151	0.590 (0.327–1.065)	0.080

AKI, acute kidney injury; HR, hazard ratio; CI, confidence interval.

Variables with *P* < 0.05 in univariate analysis and missing rate < 15.0% were entered into the analysis. All covariates entered fulfilled the assumption of proportional hazard.

## Discussion

Hyponatremia in hospitalized patients is common [1, 36], and may increase the risk of mortality in various conditions. AKI is a common and dangerous, but correctable, disease. The two clinical conditions may share various comorbidities, and there is a high chance of cross-talk among hyponatremia, AKI and mortality. Therefore, we investigated whether pre-existing hyponatremia may be associated with the development of AKI during the hospital stay. We also evaluated whether hospital acquired AKI modified the effect of hyponatremia on short-term patients mortality.

One of the major findings is that hyponatremic patients have approximately a 1.3-fold higher risk of AKI development than normonatremic patients. Sodium levels reflect on the homeostasis of water regulation in the body [1, 20]. Hyponatremia may be related to unbalanced condition of extracellular water, major organ failure or septic conditions [1, 3, 4, 37], which are some of main causes in hospital acquired AKI [13–18]. The higher rates of AKI risk factors [38] in hyponatremic patients than in normonatremic patients might affect the association between hyponatremia and AKI development. In addition, the higher levels of inflammation found in hyponatremic patients might explain the increased risk of AKI development with hyponatremia. With our study, we cannot conclude whether hyponatremia has causal relationship for the AKI development or not. However, we postulate hyponatremia might be a simple “association”, not a causative factor for AKI development since patients with persistent normonatremia showed lower risk for AKI development than patients with corrected hyponatremia. Although further study is needed to confirm this result, one thing which can be stated is that patients with pre-existing hyponatremia may need to be periodically monitored with serum creatinine levels or urine outputs for AKI development.

We observed that hyponatremia is an independent risk factor for in-hospital mortality (HR 2.481). Hyponatremia can influence on patient mortality [20]. However, if there would be some interaction between hyponatremia and AKI, these two combined pathologic conditions could contribute to dramatic increase in fatal outcomes. Since hyponatremia is tightly associated with AKI development and AKI is significantly associated with an increased risk of in-hospital mortality (HR 7.338) in our data, we examined whether there was a combined effect of hyponatremia and hospital acquired AKI on in-hospital mortality. In terms of clinical practice, it may be difficult to control patients homeostasis of electrolyte and fluid in patients with AKI [21, 22], which could be a potential explanation of this combined interaction between hyponatremia and AKI. We observed that the serum sodium level fluctuated more in patients with AKI than in those without AKI, since the AKI group showed higher rates of de novo hypernatremia than the non-AKI group by 11.6 fold. We also identified that patients with de novo hypernatremia showed a 3.297 times higher risk of in-hospital mortality than patients without de novo hypernatremia. Therefore it may demonstrate that the unstable sodium homeostasis in the AKI group could explain the combined effect between hyponatremia and AKI for in-hospital mortality.

Up to now, it has been uncertain whether patients die with or from hyponatremia. One meta-analysis suggested that the improvement of hyponatremia would revert or decrease the mortality risk related to hyponatremia [39]. However, not all the literature in the meta-analysis compared the corrected hyponatremia groups with persistent normonatremia. In fact, some studies clearly showed that the resolution of hyponatremia did not reduce the mortality risk [40–42], which is consistent with our results. Corrected hyponatremia did not reduce the risk of in-hospital mortality compared to persistent or de novo hyponatremia. The risk of in-hospital mortality between patients with corrected and persistent hyponatremia were similar. Therefore, further well-designed trials are still needed whether hyponatremia might be a bystander or a causative factor for in-hospital mortality.

There are some strengths of this study. First, the results were drawn from a very large administrative cohort. Second, all patients who admitted in this tertiary hospital were enrolled which minimized a selection bias. In addition, the clinical and laboratory data from the enrolled patients were available with low missing rate. Finally, we defined a baseline creatinine with the values 6 months before the admission which was not always possible in this large cohort study. However, the current study also has several limitations. First, we could not use glucose-adjusted serum sodium since not all patients had serum glucose assessed at the same time as serum sodium. Generally, every 100 mg/dL increase of serum glucose results in 2.4 mmol/L decrease of serum sodium levels [1]. However, the serum glucose levels of the study patients were not very high. Moreover, we used the serum glucose level as a covariate in multivariate analysis. Therefore, the effect of serum glucose on our study result should be minimal. Second, we defined AKI only by the creatinine criteria since not all patients had urine output recorded. Third, since this was retrospective, there were some uncertainties in information. We had little information on causes of AKI and death. Among patients with community acquired AKI who were excluded from this analysis, significant proportion of patients could be CKD patients. There is a chance to have other unmeasured confounders. Finally, the generalizability might be limited because this was a single-nation and single-center study.

We found that pre-existing hyponatremia may predict the development of AKI and in-hospital mortality, and that hyponatremia and AKI may lead to combined bad consequences such as in-hospital mortality, probably related to the unstable sodium homeostasis during the treatment period of hospital stay. It will be a possible option to reduce patient`s event that hyponatremic patients need to monitor the development of AKI more frequently and to avoid overcorrection of hyponatremia in AKI patients.
